# AI in music performance: the human-AI performance loop

**DOI:** 10.3389/fpsyg.2026.2001824

**Published:** 2026-10-26

**Authors:** Steven R. Livingstone

**Affiliations:** Department of Computer Science, Ontario Tech University, Oshawa, ON, Canada

**Keywords:** AI in music, AI-assisted music learning, artificial intelligence, framework, human-computer interaction, music education, music evaluation methods, music performance

## Abstract

Music is a distinctly human artform, one that has been written, taught, shared, and reinterpreted for thousands of years. The rise of powerful artificial intelligence (AI) is reshaping musical creation in ways subtle and profound. Here I propose the human-AI performance loop as a framework for understanding these developments through three principal roles: the AI listener derives musical information from sound and movement; the AI coach guides practice using performance evidence and musicians’ learning goals; and the AI partner performs with the musician in a collaborative dynamic process. With the human musician at the centre of the framework, human → AI → human exchanges become coupled through shared information and musicians’ actions. Evaluating these roles will require multilayered evidence to support their adoption: accurate and musically relevant analysis, demonstrated improvements in musician performance and knowledge through coaching, and effective and supportive coordination between musicians and AI partners. Audience beliefs about AI will also shape reception of the creative output, making musical purpose and honest representation important considerations in an AI music performance system. The human-AI performance loop provides a basis for investigating how these roles can extend support between lessons and rehearsals, broaden opportunities for ensemble performance, and contribute to musicians’ learning and enjoyment of the musical experience.

## 1 Introduction

Major advances in AI are expanding the technology’s role in music performance and learning (Mycka and Mańdziuk, 2025). While computers have long played a role in music through algorithmic composition (Fernandez and Vico, 2013; Ji et al., 2023), newer AI systems are broadening to performance assessment (Zhang et al., 2025) and improvisation accompaniment (Scarlatos et al., 2025). Together, these three applications can be viewed as operating within a recurrent musical process: compositional choices shape how musicians interpret and perform, listening and evaluation of performances generate feedback, and that feedback informs subsequent choices about composition and performance. Understanding the significance of these tools requires understanding how their outputs influence performers’ decisions, the resulting music, and listeners’ responses to the performance. Here, I introduce the human artificial intelligence performance loop, or HAIPL (/ˈhaɪ.pʊl/), as a framework that joins composition, performers, performance, feedback, listening, and interpretation to examine how AI’s participation across these relationships shapes our new musical experience.

## 2 Coupled loops of human-AI action

Three distinct roles are distinguished in HAIPL, with AI as listener, coach, and partner. Figure 1 places the human musician at the centre of these roles. An AI listener analyses auditory sound and visual movement signals, returning higher-order measurements, estimates, and comparisons of the human performance (Lerch et al., 2020; Yoo et al., 2025). An AI coach assists musicians by interpreting evidence and suggesting changes to their practice (Zhang et al., 2025). An AI partner plays music with the musician, responding in real time to shape the unfolding performance (Assayag et al., 2022), and may provide gestural cues to support coordination (Gao et al., 2024). These roles describe functions that may be combined within one or more systems.

**Figure 1:**
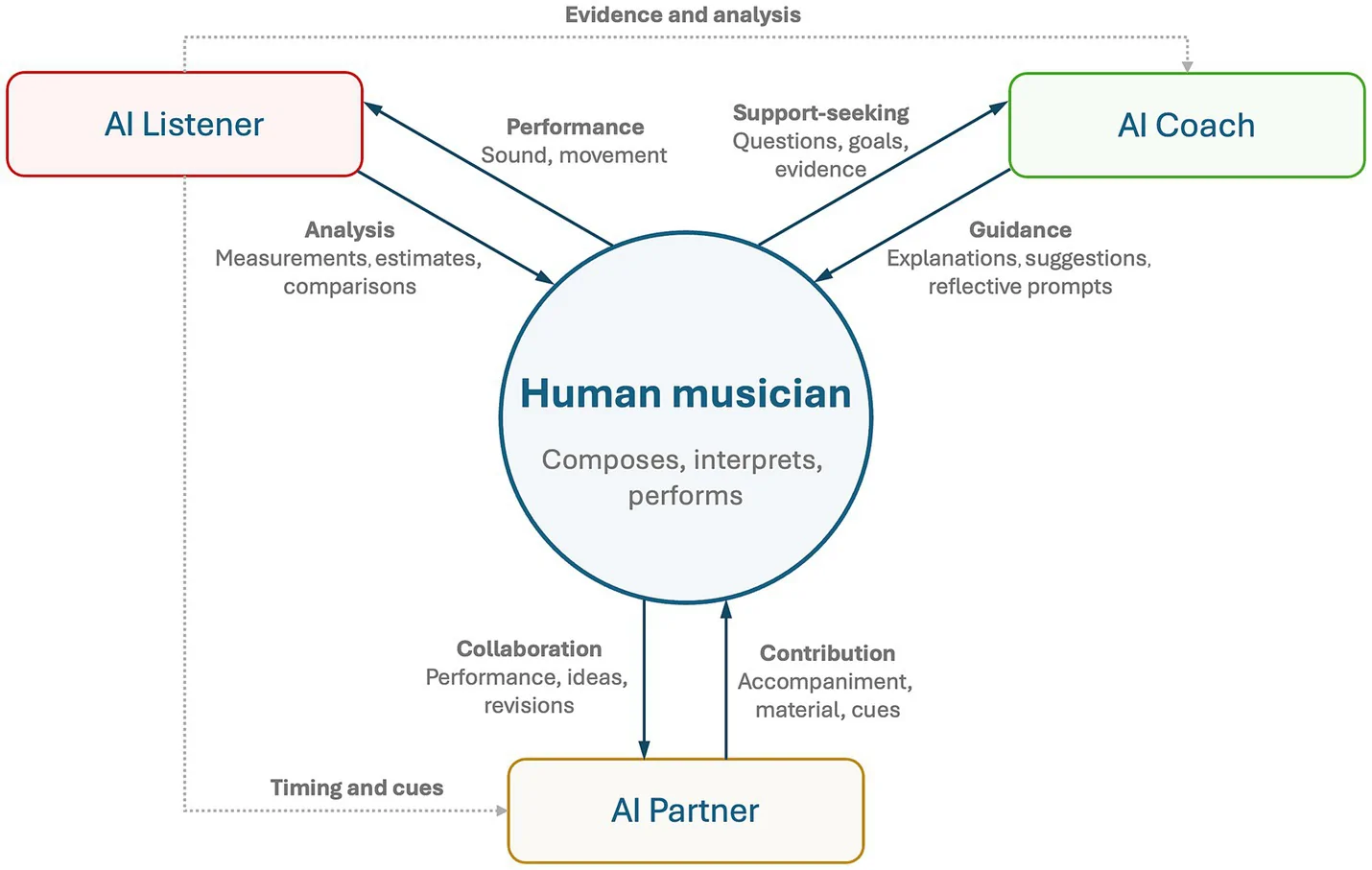
The human-AI performance loop. The framework recognises three distinct roles for AI as listener, coach, and performer that work collectively to support the human musician with measurements, guidance, and sound.

The arrows within the framework represent information exchange between the musician and the AI system. In the AI listener exchange, sound and movement are converted into evidence the musician can use, such as timing measurements for comparing two renditions (Lerch et al., 2020). The AI coach supports the musician through guidance: a question or practice goal frames how evidence is interpreted by the AI, and which changes it suggests for composition and performance. Li et al. (2025) describe learners reporting practice difficulties and receiving AI-generated guidance within a structured reflection process. The AI partner interaction represents an exchange of musical contributions: in ReaLJam, the musician’s melody affects the generated chords, which provide a harmonic context for further melodic choices (Scarlatos et al., 2025). Information can also pass between AI functions, indicated by dotted arrows. An AI listener’s analysis can supply evidence for a coach’s explanation, while its timing estimates could ‘cue’ a partner’s musical entry. These connections allow analysis to inform both guidance to the musician and contributions to the music performance.

A loop is defined as a recurrent human → AI → human exchange: the musician provides input, receives an AI response, and the musician uses this feedback to shape subsequent musical activity. Loops become coupled when one exchange influences another. In a theoretical system that supports musician practice, the AI listener could analyse the performance continuously and share timing information with both the AI coach and partner. The AI coach could use this evidence to discuss rushing and suggest slowing the practice, while the AI partner uses it to coordinate the accompaniment. If the musician adopts a slower tempo, the AI partner could adjust dynamically, producing a modified performance that would in turn provide new input to the AI listener. These loops may operate concurrently or sequentially, becoming coupled through the system’s design and the musician’s decision process.

## 3 Analysing musical performance with an AI listener

The AI listener acts as the signal analyst within HAIPL. It collects raw auditory–visual information and uses machine-learning models to estimate higher-order features such as pitch, timing, dynamics, movement velocity and acceleration (Lerch et al., 2020; Yoo et al., 2025). These analyses can be returned to musicians in different modalities – numerical, graphical, sounded, or pictorial - helping the musician to examine specific passages or compare performances. The AI listener is a bedrock of the framework, supporting other computational functions: its analysis provides evidence for an AI coach’s guidance, while its timing estimates can help an AI partner coordinate its own musical contribution.

Two systems illustrate the range of information an AI listener can provide. Huang et al. (2020) combined pitch contours and score information to predict expert ratings of note accuracy, rhythmic accuracy, and musicality in student wind performances. Including the score improved assessment predictions by providing information about the music being performed. The VioPose system combined sound and visual information to estimate violinists’ movement over time, including details relevant to bowing and vibrato (Yoo et al., 2025). Together, these tools illustrate how AI analysis can connect an audiovisual signal with its notated material and the physical actions a musician uses during performance.

For AI listeners to see wider adoption, their outputs need to be validated. Inaccurate measurements may mislead musicians, disrupt an AI partner’s timing, or provide an unreliable basis for AI coach guidance. Validation would ideally compare system outputs with reference measurements of recordings not used to train or tune the listener system. For example, vibrato detection could be evaluated against expert annotations by quantifying missed events, false detections, and errors in onset and offset timing. Kim and Song (2026) showed that vibrato in an archival sanjo recording required manual segmentation after automatic methods failed to identify its boundaries reliably. Validation should cover the target instruments and recording conditions, with acceptable error thresholds known to the musician that allows them to make informed decisions on when to follow or possibly disregard AI feedback.

While an AI listener can reveal how a musician’s timing, dynamics, or articulation differ from a reference performance, these differences should not automatically be treated as errors. Peter et al. (2023) examined reconstruction-error measures used to evaluate generative models of expressive piano performance. Their findings highlighted how comparisons depend on the chosen reference performance and can miss differences that listeners hear. For musicians, such analysis can draw attention to passages worth revisiting without prescribing a particular interpretation. For example, a slower phrase ending may reflect a deliberate expressive choice (Demos et al., 2020; Guichaoua et al., 2024). The musician must therefore decide whether a measured difference represents a problem to correct or an expressive choice to retain.

At its core, an AI listener converts low-level auditory and visual signals into musical information, such as the tempo of a performance, accuracy of played pitches, and performed loudness relative to notated dynamics. The value of these measurements depends on their accuracy and relevance to the musical task. These higher-level measurements provide evidence to musicians about how their performance is unfolding, while acting as the sensory module for a broader network of AI models.

## 4 Independent musicianship with an AI coach

The AI coach assumes an instructional role within the framework. Like a music teacher, the AI coach uses observations from a musician’s performance, together with higher-level instructional goals, to guide what and how the student practises. The AI coach can explain a difficult passage, suggest an exercise strategy, and review whether subsequent attempts show improvement. Its evidence may come from the AI listener’s analysis, the musician’s own description of a problem, or both. Like the AI listener, the system must offer value to the musician. For adoption of this technology to grow, the AI coach’s value must be shown improve the performance and musical knowledge of musicians.

Two recent studies illustrate different ways of providing and evaluating instructional guidance. LLaQo combines listener and coach functions in one system, using audio analysis to support written feedback about piano performances and suggestions about what the student should practise. A study with 20 students and teachers rated its feedback more favourably than those of comparison models on relevance to the questions and correspondence with the performances. However, the study did not test whether acting on this guidance improved students’ playing (Zhang et al., 2025). Li et al. (2025) evaluated a six-week randomized vocal-training intervention in which learners used Tencent (2024) Yuanbao chatbot to discuss practice difficulties and receive suggested adjustments. AI dialogue was combined with recording comparison, reflective journals, and instructor input. The intervention increased students’ overall scores on a self-report questionnaire covering practice planning, monitoring of progress, and use of learning strategies. While singing scores increased in both groups, the intervention produced no significant improvement over the control group on independently rated performance measures.

Other AI coaching systems have shown improvement in student performance outcomes. Violy combines real-time visual pitch feedback with reports on intonation, rhythm, and note accuracy, coupled with demonstration videos and accompaniment (Ou et al., 2025). In a four-month study involving 40 violin majors, students using Violy showed larger gains in performance examination scores than the control group. Examinations were assessed by an expert panel unaware of the students’ group assignments. Students using Violy also reported greater confidence in performing, with increases in independently assessed performance outcomes. While a a-prior power analysis was conducted, the study did not report random assignment or preregistration, and the app combined several forms of practice support, leaving the specific contribution of its AI feedback unresolved.

For an AI coach to be useful during practice, its guidance must be relevant, comprehensible, and timely. How and when feedback is delivered is an important design choice – a verbal explanation may disrupt auditory attention while pictorial movements may compete for visual-motor attention. In a randomized controlled study of violin beginners, Blanco et al. (2021) found that real-time visual feedback on sound and bow movement improved bow straightness during practice, while sound stability tended to worsen when feedback was displayed. After feedback was removed, the feedback group showed greater improvement in loudness stability than controls. Although this study examined augmented feedback rather than AI coaching, the findings illustrate how feedback can introduce competing demands during practice while benefiting subsequent performance. An AI coach should therefore match the timing and context of guidance to the task, allowing musicians to try a suggestion and hear its effect before receiving further advice.

For AI coaching systems to see wider adoption, they must demonstrate that their guidance improves musicians’ performance and musical knowledge, both in the short and longer term. Performance assessments pre- and post- intervention should use independent raters blinded to group assignment and study hypotheses. Where feasible, studies should randomly assign participants to conditions with comparable practice time, teacher contact, and other support, while controlling for relevant covariates. Establishing these benefits will require rigorously designed studies, alongside evaluation of the costs of providing such support. The potential educational contribution though is substantial: effective and affordable AI coaching could make individual practice guidance more widely available, extending the support musicians receive between lessons and providing a valuable supplement to standard music education.

## 5 Playing together with AI as partner

The AI partner takes a performing role within HAIPL, playing music alongside the human musician. It may provide improvised accompaniment or perform a dedicated part in a duet or larger ensemble, take turns with the musician, or transform the musician’s sound during performance. Information from the AI listener’s analysis is central to the partner’s function, helping it to adjust its timing and musical interpretation in response to the musician’s playing and gestural cues. The musician, in turn, responds to what the partner plays, shaping the performance through coordinated joint attention as in human duetting performance (Goebl and Palmer, 2009). The value of an AI partner therefore depends on whether musicians can coordinate with and influence how it plays, to create a rewarding performance.

Earlier computational rule-systems allowed users to modify musical expression in real-time through changes to score and performance features (Livingstone et al., 2010), while newer AI systems can more flexibly adapt their playing in response to a musician’s performance. Two such systems illustrate how this interaction can support improvised accompaniment and the performance of prepared repertoire. ReaLJam generates accompanying chords in response to a musician’s melody. Testing found that musicians generally enjoyed playing with the system, and valued its unexpected musical ideas, while noting limitations in control over harmonic choices and the system’s recognition of phrase structure (Scarlatos et al., 2025). ACCompanion performs a prepared accompaniment that responds to the soloist’s tempo, dynamics, and articulation. The authors reported that the system could respond to expressive changes in timing and dynamics but could also start too fast or reinforce unintended slowing, making it difficult to stay together (Cancino-Chacón et al., 2023). Together, these findings suggest that AI partners can support rewarding musical exchanges, although coordinating and shaping the performance remains a challenge.

AI partnership can also involve visible coordination cues and several artificial players within a single performance. Physical gestures can help musicians coordinate during ensemble performance (Chang et al., 2017) and reflects emotional expression (Chang et al., 2019). In a study with the robot Shimon, programmed head gestures helped pianists synchronize with tempo changes in its preprogrammed accompaniment (Gao et al., 2024). Multiple agents offer another way to extend the partnership, contributing different parts within a larger ensemble. Brade et al. (2026) described a saxophonist performing with 24 Somax2 agents (Assayag et al., 2022) drawing on precomposed musical material, while a second human performer managed transitions between sections. The arrangement allowed one musician to interact with several artificial contributors while retaining human direction over the performance’s larger structure.

The usefulness of an AI partner depends partly on what the musician is trying to achieve. An accompaniment that is predictable may support a musician’s practice of a prepared passage, while an unexpected contribution may stimulate improvisation. In a comparison with iReal Pro, a backing-track generation app, musicians more often preferred EAR Drummer’s reactive accompaniment for stage performance than for practice, illustrating how an appealing musical response can also become a distraction (Ostermann et al., 2021). In a study of Living Looper, a neural audio looping system, the violinist experienced a recurring hiss as disruptive in one context but incorporated it as drone material in another (Stefánsdóttir and Magnusson, 2025). This latter example highlights the musician’s ability to recover creatively from system failure. Musicians therefore need ways to shape, redirect, or reject a partner’s contributions while retaining the freedom to incorporate unexpected musical ideas.

To fully evaluate the effectiveness of an AI partner, research should follow musicians across repeated performances, assessing whether they can coordinate entries and tempo changes, shape musical direction, and recover from unexpected responses. Recordings and musicians’ accounts can show whether the partnership supports their musical intentions, while comparisons with fixed accompaniment can help identify what AI partner responsiveness adds. When systems are designed as practice partners, evaluation could test whether the experience improves subsequent performance with human musicians. Ultimately, the real test of AI partners is whether musicians continue to use the systems once research studies are concluded.

The potential contribution of AI partners is substantial. The technology could broaden the repertoire musicians can explore by supplying additional parts for duets, trios, and larger ensembles. For musicians who normally perform in groups, AI partners could provide practice between rehearsals, allowing them to work on their part and the surrounding music in an individual setting. Reliable and responsive AI partner systems could therefore enrich both a musician’s playable library and the opportunities available for performing with others.

## 6 Discussion

HAIPL captures the many ways that AI is being integrated into music performance. The framework connects research on performance analysis, music learning, and interactive performance by following how AI can influence musician activity. Its roles have precedents in accounts of computers as coaches and colleagues (Lubart, 2005) and musical agents with different tasks and forms of interaction (Tatar and Pasquier, 2019). COFI and the user-centred framework of Moruzzi and Margarido (2024) describe how communication, participation, and control shape creative collaboration (Rezwana and Maher, 2023). Bown’s (2021) account of distributed creativity further explains why outcomes must be understood through relationships among people and technologies. HAIPL builds on these perspectives by tracing consequences across analysis, instruction, and co-performance. A listener’s timing estimates may shape accompaniment, while a coach’s interpretation of the resulting coordination may reinforce the musician’s developing habits. Following these connections enables researchers to examine whether improvements within one exchange support the musician’s broader goals, including subsequent performance with human partners. The framework therefore directs attention to consequences that evaluating analysis, guidance, or accompaniment separately may miss.

For these systems to see wider adoption, their benefits for musicians will need to be established through rigorously designed interventions, including parallel-arm randomised controlled trials (Hopewell et al., 2025). For AI partners, trials could compare practice with and without AI accompaniment, asking whether it improves subsequent performance with human partners, assessed through accuracy measures and independent listener ratings. For AI coaches, trials could compare practice with and without AI guidance, using standardised educational assessments to establish whether coaching improves performance skills and musical knowledge, and whether gains persist after support is withdrawn.

Longer-term evaluation should also consider how feedback cycles may shape learning over repeated practice sessions. For a pianist learning a duo part, AI listener analysis can guide an AI partner’s accompaniment. The pianist adjusts to this response, supplying new input for subsequent exchanges. Repeated coordination with the AI partner may reinforce timing habits specific to the AI partner. If an AI coach interprets smoother coordination as progress, its guidance could further reinforce these habits. The eventual human partner may respond differently, requiring the pianist to revise those expectations. The AI listener’s analysis could be accurate and the AI partner’s accompaniment well-coordinated, yet the AI coach could reinforce habits that hinder later performance with human partners. This raises a specific research question: how does coaching that reinforces coordination with an AI partner affect the musician’s subsequent adaptation to a human partner?

Knowing that AI has been used in the musical process shapes an audience’s experience. Ansani et al. (2025) found that identical piano audio was liked less when paired with an apparently AI-driven, self-playing piano than a visible pianist. These responses are not uniform (Shank et al., 2023), but Dunham et al. (2025) found stronger negative responses to AI-attributed music among listeners who regarded creativity as uniquely human. These results occur alongside wider public concern about AI’s role in creative work. A recent survey conducted by Ipsos for Deezer found widespread concern that AI could reduce musical creativity, threaten artists’ incomes, and must be clearly identified to listeners (Wendel, 2025). Thus, technical capability alone should not determine AI’s place in musical performance. Musicians and educators must consider whether its involvement serves broader goals and what it changes about the human contribution to music making. Users must ultimately decide if AI is best used as background support or as a featured performer, while representing its contribution honestly so audiences can understand how the music was made.

The human-AI performance loop (HAIPL) brings analysis, instruction, and collaboration into a common framework for studying how AI shapes musical activity. The AI listener can help musicians examine and understand their performances; the AI coach can extend individual practice guidance between lessons; and the AI partner can broaden the repertoire musicians explore and their opportunities for ensemble performance. The value of this framework will depend on research that measures how these roles support and enrich musical performance.

## Data Availability

The original contributions presented in the study are included in the article/supplementary material, further inquiries can be directed to the corresponding author.
